# On-job training program for food handlers about food safety standards

**DOI:** 10.1186/s12889-026-26228-4

**Published:** 2026-03-11

**Authors:** Suzan Mohamed Hamdy Ibrahim, Magda Abd El-Sattar Ahmed, Ghada Sobhy Hassan

**Affiliations:** 1 Department of Community Health Nursing, Faculty of Nursing, Ain Shams University, Cairo, Egypt; 2 Egypt Health Care Authority, Cairo, Egypt; 3 Nursing Department, North Private College of Nursing, Arar, Saudi Arabia

**Keywords:** Food handlers, Food safety standards, Hospital kitchen, And on-job training program

## Abstract

**Background:**

Food safety training serves as a link between research evidence informing safe food handling practices and implementation by food handlers. Healthcare is a setting where foodborne outbreaks can cause considerable morbidity and mortality. Therefore, this study aimed to assess the effect of an on-the-job training program about food safety standards on food handlers’ knowledge and practices in a hospital kitchen.

**Methods:**

A quasi-experimental one-group pretest- posttest design was conducted at the Egypt Health Care Authority Hospitals located within Port Said Governorate. The study sample comprised 70 hospital kitchen food handlers both male and female, and the study lasted for six months. Data collection was performed utilizing two tools: a structured interviewing questionnaire to assess food handlers’ level of knowledge on food safety standards and an observational checklist to evaluate their practices. Analysis of data was done using the chi-square test or Fisher’s exact test, logistic regression and Pearson’s correlation at a significance level of *p* < 0.05.

**Results:**

Posttest, 60% of food handlers had a good total knowledge level compared to 25.7% before; the program significantly improved food handler knowledge and observed practices related to food safety standards (*p* < 0.001). Adequate practices rose from 60% to 84.3% with a statistically significant difference (*p* < 0.001). Data were analyzed by chi-square test or Fisher’s exact test followed by advanced regression analysis. A significant correlation existed between knowledge and practices regarding food safety standards immediately after the program.

**Conclusion:**

Based on the results, the current study findings proved that the application of an on-the-job training program about food safety standards improved food handlers’ knowledge and practices about food safety standards. Therefore, implementing an on-the-job training program for food handlers in hospitals significantly enhances their understanding of food safety standards and practices application, consequently, reducing the risk of foodborne diseases and fostering a culture of continuous improvement.

**Supplementary Information:**

The online version contains supplementary material available at 10.1186/s12889-026-26228-4.

## Background

Food is essential for human survival; however, it may pose serious health risks when contaminated with biological, chemical, or physical hazards. Food is considered safe only when the food is free from such hazards [1]. In addition, food allergens have become an increasingly important cause of food-related morbidity and mortality, as even small quantities may trigger life-threatening reactions, specially in healthcare settings serving vulnerable populations [2].

According to the World Health Organization, contaminated food causes approximately 600 million illnesses and 420,000 deaths worldwide each year [3]. Food handlers play a critical role in preventing food contamination; however, inadequate training continues to compromise food safety practices. In the European Union, only 37% of employees receive adequate food safety training, contributing to persistent knowledge gaps. Beyond Europe, the Poison Control Center of Ain Shams University, Egypt, conducted a retrospective study in which, of the 1748 cases of food poisoning admitted in early 2010, the majority were of the “accidental poisoning” type and the principal cases were food poisoning and poisoning by organophosphate-contaminated insecticides [4].

Food safety refers to the conditions and measures necessary to ensure that food remains safe throughout handling, preparation, and storage [5]. Adherence to food safety standards is crucial for the prevention of both direct and indirect contamination and to protect public health [6].

The food we eat, which is also a greater source of microorganisms, result in more deaths than other diseases that include HIV/AIDS, malaria and measles [7]. Each year, almost 48 million Americans suffer from illnesses nationally, resulting in many hospital stays and deaths from foodborne illnesses. The number of diseases associated with contaminated food is over 200 [8]. The Centers for Disease Control and Prevention (CDC) states that food handlers are the cause of approximately 20% of the infections that are directly related to food [9].

Food poisoning from hospitals is especially problematic because of the already fragile health status of the patients. That makes it more difficult for hospital patients who are not in good health to deal with the health challenges [10]. The risk of illness due to pathogens that thrive from poor food handling is especially great in people with weakened immune systems, also contribute to these outbreaks [11, 12].

The Middle East and North African region which includes Egypt is categorized as the 3rd greatest rated burden of foodborne diseases per capita. Each year, about 100 million individuals in these areas suffer from illnesses resulting from the consumption of contaminated food. In Egypt, the foodborne illness outbreaks can also be attributed to poor food handling in food services [13, 14]. In Egypt, a major food poisoning outbreak in 2018 resulted in high hospitalization rates and fatalities, highlighting the severe implications of inadequate food safety practices [15].

Since 2013, some research has aimed to assess the dietary services staff’s knowledge using self-reported surveys, while the lack of data concerning actual practices has been problematic. Some of these knowledge-based interventions have had limited, if any, impact on practices [16, 17]. This research differs in that, in addition to measuring knowledge, direct observation was also conducted in a select number of hospital kitchens to document simultaneous evidence specific to the relevant context of low- and middle-income countries.

Thus, the aim of this study became to assess the impact of an on-the-job training program for food handlers on their knowledge and practices as it pertains to the food safety standards. It is the expectation of the research that the food handlers will demonstrate improved knowledge and application of the food safety standards immediately following the training program.

## Methods

### Study design, period and area

A single-group quasi-experimental design with pre- and post-test assessments was used for this evaluation. This design was necessary because the intervention was implemented as a hospital-wide quality improvement initiative in the Egypt Health Care Authority kitchens in Port Said Governorate. Ethical and administrative considerations, specifically the risk of withholding food safety training from food handlers, which could expose hospitalized patients to preventable foodborne illnesses, made it unfeasible to include a control group. Such designs are commonly applied in public health and healthcare settings to assess the operational feasibility of training programs. The evaluation was conducted over a six-month period, from September 2022 to February 2023.

### Sampling size and technique

The study sample comprised 70 hospital kitchen food handlers, including both male and female individuals. A convenience sampling technique was utilized. The sample size was determined based on feasibility and workforce availability during the study period. All eligible food handlers working in the selected hospital kitchens who were available during data collection were invited to participate. Given the applied nature of the intervention and the fixed number of food handlers employed in the study setting, a formal a priori power calculation was not conducted.

### Data collection tools and techniques

Data were collected using a structured interview questionnaire and an observational checklist, both developed by the researcher based on a thorough review of relevant literature and they were used both before and immediately after the on-the-job training sessions [18, 19]. The structured questionnaire included two sections: the first captured participants’ demographic and work-related characteristics, including age, gender, education level, and work experience. The second section assessed prior food safety training, health certifications, and immunization status, alongside 49 multiple-choice questions evaluating food safety knowledge across four domains: foodborne illness (10 items), food hygiene (11 items), safe food handling (16 items), and prevention of food contamination (12 items). Each correct answer received a score of one, while incorrect or unclear responses received zero. Knowledge scores were expressed as percentages and classified as good (≥ 75%), average (50–74%), or poor (< 50%).

The observational checklist, designed according to national and international food safety standards, measured procedural compliance of food handlers before and immediately after the training. It included 46 items across two main areas: hygiene-related practices (27 items) and food production processes (19 items). Hygiene practices were further subdivided into outfit compliance (3 items), hand hygiene (8 items), and cross-contamination prevention (16 items). Food production processes were divided into raw material receipt (5 items), food preparation (3 items), defrosting (4 items), thermal processing (3 items), and final distribution (4 items). Each correctly performed step was scored one, while errors received zero. Overall compliance scores were converted to percentages and categorized as adequate (80–100%) or inadequate (< 80%(.

### Ethical clearance and pilot study

The Ethics and Research Committee of Ain Shams University’s Faculty of Nursing approved the study (approval code 25.08.793). Each participating food handler gave their valid informed consent after being made aware of the study’s strict confidentiality protections and their right to withdraw at any time. The clarity, objectivity, feasibility, application, and general practicality of the used data-gathering tools were then assessed in a pilot study with 10% of the target sample. The average length of each session was also noted. Following analysis of the pilot results, necessary modifications such as removals, edits, additions, and revisions were made to produce an optimal version of the instruments prior to extensive data collecting.

### Instruments validity and reliability

The instrument validity was evaluated by a jury of five experts from the community health nursing department, Faculty of Nursing, Ain Shams University, following a recommendation from the scientific medical advisory committee for format, layout, accuracy, and consistency of the tools. While the reliability of the instrument was tested by Cronbach’s alpha test of reliability (α = 0.8), and the tool proved to be strongly reliable.

### Training program and trainer intervention

A systematic strategy was used to guarantee that the trainer intervention and training program were consistent across all sessions. A pilot test was implemented before the sessions to ensure consistency and uniformity. In addition, all the training sessions were conducted by the researcher, who used the same session plan throughout, the same training session employed identical training aids, and all provided the same training materials.

A comprehensive, detailed on-the-job training program was provided to all food handlers who were part of the study. The program consists of five training sessions, each lasting 45-minutes. The training was provided in an operational situation, and in order to ensure that the training was appropriate, it was compressed in order to cover all of the essential points.

This multifaceted approach has provided training about basic terminology in food safety, classifications of microbial and chemical risks and the epidemiology of foodborne disease. It included hand hygiene, personal hygiene, temperature control, and prevention of cross-contamination, coupled with instruction on structured food safety procedures. Reinforcement of important concepts included the distribution of additional handouts. Scripting of role-play exercises, guided interactions, lectures, and participatory demonstrations were used to foster development of procedural knowledge required to support sustainable practice of food safety .

### Statistical analysis

The research data was retrieved from the National Center for Education Statistics (NCES) and processed in the Statistical Package for Social Sciences (SPSS) version 23.0 (IBM Corp., Chicago, IL, USA). For the nominal level/ categorical variables, we calculated the total and the percentages corresponding to the total from each country. For the continuous/ scale variables, we calculated the means and standard deviations (SD). To assess relationships between the categorical variables, the chi-square test of independence was used, while Fisher’s exact test was used when expected counts in cells of the table were five or fewer. Knowledge and practice scores were assessed using the Pearson’s correlation coefficient. Change in knowledge and practice scores were assessed using multiple linear regression whereas factors associated with achievement of adequate practice levels (≥ 80%) were assessed using binary logistic regression. Statistically significant p-value thresholds of 0.05 and 0.001 or less were used in the analyses to indicate highly statistically significant cases.

## Results

Table 1. The analysis showed that 37.2% of the food handlers were in the age category of 30–40 years, with a mean ± SD of 35.33 ± 9.27. Among the food handlers, 82.9% were female, 40.1% had completed their diploma level of education, and only 15.7% held a bachelor’s degree. Additionally, 32.9% worked as food providers, and 45.7% had work experience ranging from 1 to less than 5 years.

**Table 1:** Characteristics of the study population (*N* = 70)

	No.	%
Demographic data		
Age “years”		
20 - <30 years	20	28.6
30 - < 40 years	26	37.2
40 - < 50 years	19	27.1
≥ 50 years	5	7.1
*Mean ± SD (age)*	35.33 ± 9.27	
Gender		
Male	12	17.1
Female	58	82.9
Years of experience “years"		
< 1 year	6	8.6
1- < 5 years	32	45.7
5- < 10 years	16	22.9
10 - < 15 years	8	11.4
≥ 15 years	8	11.4
Educational Qualification		
Primary	1	1.4
Preparatory	4	5.7
Secondary	26	37.1
Diploma	28	40.1
Bachelor’s degree or higher	11	15.7
Working activity (job)		
Nutrition Specialist	8	11.4
Food technician	15	21.4
Chef	11	15.7
Food providers	23	32.9
Assistant chef	11	15.7
Health inspector	2	2.9

Table 2. According to the result 51% of the food handlers did not receive training courses on food safety. Additionally, 91.4% had health certificates, and 100% were vaccinated.

**Table 2:** Distribution of the studied food handlers according to their training, health certificate, and vaccination status (*N* = 70)

Items	No.	%
Trained	34	49
Certified	64	91.4
Vaccinated	70	100.0

Table 3. The result showed that immediately following the on-the-job training program, 58.6%, 60%, 61.4%, and 60% of participants had good levels of knowledge regarding safe food handling, food hygiene, foodborne diseases, and prevention of food contamination, respectively. In comparison, before the program, in Table 3, there were 21.4%, 25.7%, 31.4%, and 28.6% respectively. The highest score for good knowledge was related to foodborne diseases. There was a statistically significant difference (p-value < 0.001**) when the total level of knowledge regarding safe food handling before and after the program was compared.

**Table 3:** Distribution of the studied food handlers according to their total level of knowledge about food safety standards (*N* = 70)

Items	Pre-Program						Post-Program						test	P_value ^a^
	Good		Average		Poor		Good		Average		Poor			
	No.	%	No.	%	No.	%	No.	%	No.	%	No.	%		
Safe food handling	15	21.4	40	57.1	15	21.4	41	58.6	25	35.7	4	5.7	17.714*	< 0.001**
Food Hygiene	18	25.7	37	52.9	15	21.4	42	60.0	22	31.4	6	8.6	8.480**	0.011*
Food-borne diseases	22	31.4	28	40.0	20	28.6	43	61.4	19	27.1	8	11.4	2.465**	0.163
Prevention of food contamination	20	28.6	32	45.7	18	25.7	42	60.0	20	28.6	8	11.4	3.897**	0.048*
Total	18	25.7	35	50.0	17	24.3	42	60.0	23	32.9	5	7.1	18.628*	< 0.001**

*Used Fisher’s exact test for values less than 5 - ** Used Chi-Square test

Additionally, before the on-the-job training program, 25.7% of the studied food handlers had a good level of knowledge, 50% had an average level, and 24.3% had poor knowledge regarding food safety standards. However, immediately following the training program, 60% of them had good knowledge, approximately 33% had an average level, and only 7% had poor knowledge. There was a statistically significant difference (p-value < 0.001**) when the total level of knowledge regarding food safety standards before and immediately after the program was compared.

Table 4. Shows that 84.3% of food handlers had an adequate total level of practices regarding food safety standards immedialetly after the on-the-job training program, whereas 60% had an adequate total level of practices before the program, with a statistically significant difference (*p* = 0.003*) when the total level of practices before and after the program was compared. Furthermore, this table shows that 87.1% of the food handlers had adequate total practices regarding food hygiene immediately after the on-the-job training program, whereas 54.3% had adequate total practices before the program, with highly statistically significant differences (*p* < 0.001**) when compared before and immediately after the program. Additionally, 82.9% of them had an adequate total level of practices related to their course of work, with statistically significant differences (*p* = 0.021*) when compared before and after the program.

**Table 4:** Distribution of studied food handlers according to their total level of practices toward food safety standards, which include the following items (food hygiene and course of work in the kitchen) (*N* = 70)

Level of practices	Pre-Program				Post-Program				x2	P_value ^a^
	Adequate		Inadequate		Adequate		Inadequate			
	No.	%	No.	%	No.	%	No.	%		
Food hygiene	38	54.3	32	45.7	61	87.1	9	12.9	16.694	< 0.001**
Course of work	45	64.3	25	35.7	58	82.9	12	17.1	5.290	0.021*
Total level of practices	42	60.0	28	40.0	59	84.3	11	15.7	9.099	0.003*

**Fig. 1:**
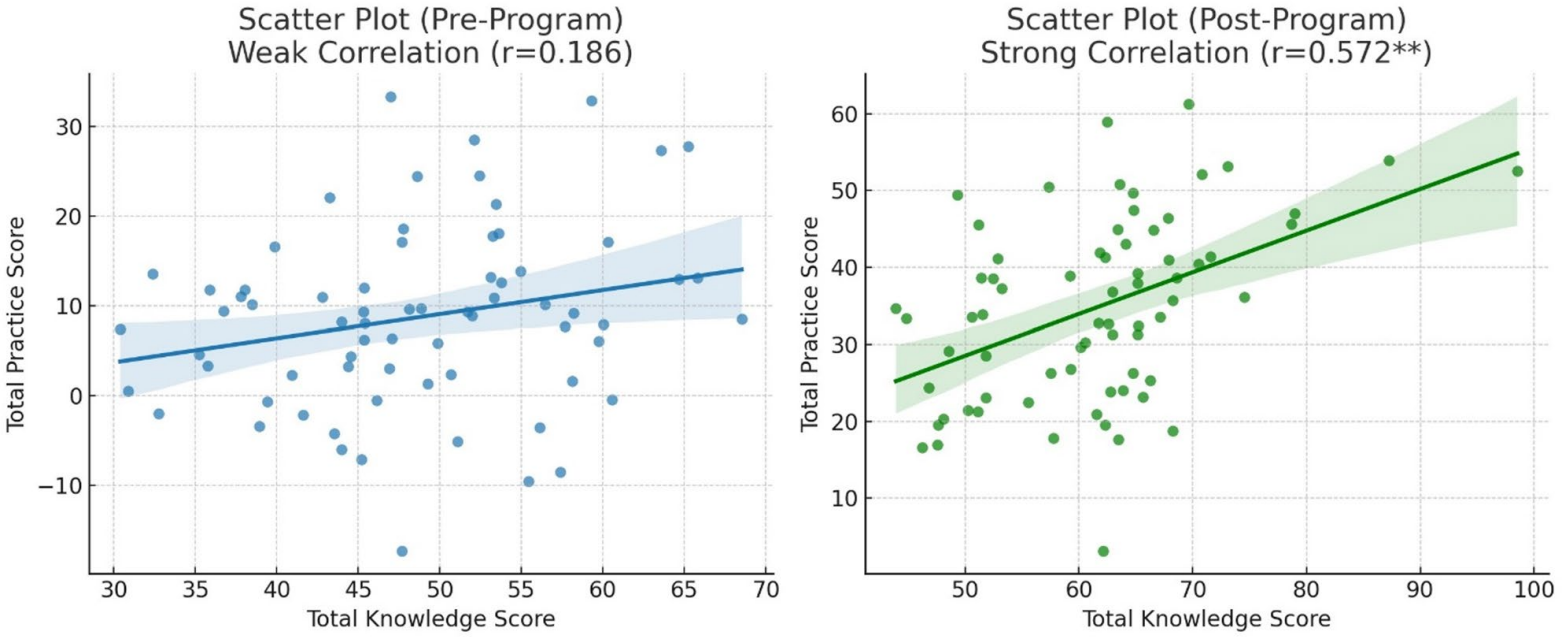
Correlation matrix between food handlers’ total level of knowledge and total level of practices related to food safety standards

Table 5 & Fig. 1. Show that there was a highly significant correlation (*p* < 0.001**) between the total level of food handlers’ knowledge and the total level of practices regarding food safety standards immediately following the implementation of the on-the-job training program. However, before the program, there was no significant correlation between the total level of food handlers’ knowledge and the total level of their practices regarding food safety standards.

**Table 5:** Correlation matrix between food handlers’ total level of knowledge and total level of practices related to food safety standards (*N* = 70)

	*r*-value (Pre-Program)	*p*-value (Pre-Program)	*r*-value (Post-Program)	*p*-value (Post-Program)
Total score of knowledge	0.186	0.582	0.572	< 0.001**
Total score of practice				

**p-value < 0.001 is highly significant

Table 6. The regression model showed a good fit with an R-squared value of 0.844, which means that around 84.4% of the variability in the change of total knowledge was captured by the dependent variables in the model. The model as a whole was significant (F-statistic = 44.79, *p* < 0.001).

**Table 6:** Multiple linear regression predicting change in total knowledge (*N* = 70)

Variable	B (coef)	SE	t	*p*-value
Gender	0.6466	0.639	1.012	0.316
Age	-0.0307	0.039	-0.784	0.436
Exper	0.0196	0.052	0.380	0.705
Educ	-0.2321	0.327	-0.709	0.481
Job	-0.2382	0.212	-1.125	0.265
Train	-0.0756	0.641	-0.118	0.906
H. cert	16.9629	1.295	13.094	< 0.001
Vaccin	16.9629	1.295	13.094	< 0.001
Total_Knowledge_Pre	-0.6585	0.044	-15.088	< 0.001

The results strongly support the notion that the training program was successful in improving the food handlers’ knowledge. The strong positive relationship between the health certificate and vaccination status, and knowledge gain, suggests that individuals who already appreciate health regulations are more likely to be well-trained and benefit more from such programs. This may be due to an already heightened awareness of health issues or an inherent positive inclination to comply with safety standards.

On the other hand, the negative relationship between the pre-program total knowledge and the change in knowledge was equally telling of the knowledge gap the program has successfully addressed and the agile learning attitude of the participants who needed it the most. This confirms that the training program succeeded in diverse levels of understanding of the participants, and especially among those with lower initial scores.

Health certificate status and vaccination status were highly correlated variables in this sample, as nearly all participants possessed both. Their similar regression coefficients reflect this overlap rather than independent effects.

Tables 7 and 8. Results from the multiple linear regression analysis showed that knowledge score and prior training completed before the program significantly predicted practice performance after the program. More specifically, the practice score increased by 0.21% for every 1% increase in knowledge score (*p* = 0.038), demonstrating that greater knowledge led to an enhancement in the performance of food safety practices. Prior training showed the most significant effect. Participants with prior training scored, on average, 65.36% points higher in practice compared to those without previous training (*p* = 0.002). Age, gender, education, and years of experience were not significant predictors, indicating that these demographic factors did not bias the training.

**Table 7:** Multiple linear regression predicting change practice scores (*N* = 70)

Predictor	B	SE	t	*p*
knowledge score pre	0.212	0.1	2.118	0.038
age	-0.047	0.086	-0.55	0.585
Gender	0.248	1.516	0.164	0.87
Educ	0.744	0.761	0.977	0.332
Exper	-0.017	0.112	-0.147	0.884
Train	65.363	9.252	7.065	0.002

**Table 8:** Binary logistic regression predicting adequate practice (≥ 80%) (*N* = 70)

Predictor	B	SE	z	*p*	OR
knowledge score	0.113	0.077	1.462	0.144	1.119
age	-0.018	0.082	-0.219	0.826	0.982
Gender	-0.035	1.292	-0.027	0.978	0.966
Educ	1.605	0.868	1.849	0.065	4.979
Exper	-0.066	0.115	-0.574	0.566	0.936
Train	-11.849	7.98	-1.485	0.138	0.001

In the binary logistic regression model, the knowledge score continued to be associated, if not significantly (*p* = 0.144), with an increased likelihood of achieving adequate practice (≥ 80%) with OR = 1.12. Education level showed a borderline significant effect (*p* = 0.065), with participants of higher educational level almost 5 times more likely to achieve adequate practice compared to those with lower educational levels. The model also showed no significant association with age, gender, experience, or prior training. This represents that knowledge and education improve practice, though the benefits are likely more visible when practice is considered on a continuum rather than a binary pass/fail. The improvements were assessed immediately after training. No long-term follow-up was conducted to determine sustainability, which is acknowledged as a study limitation.

## Discussion

The research is particularly important for public health and patient safety, as hospital patients are highly vulnerable to foodborne illnesses. The training program effectively improved food handlers’ knowledge and practical adherence to food safety standards, therefore reducing the risk of foodborne outbreaks, ensuring compliance with established regulations, and enhancing overall food handling practices. These findings highlight the critical role of targeted training in improving food safety preparedness within high-risk healthcare settings.

### Demographic features of the study sample

The study included 70 male and female food handlers working in hospital kitchens of the Egypt Health Care Authority Hospitals in Port Said Governorate. Sociodemographic data encompassed age, sex, education, work experience. Compared with other studies in Lebanon (2018) and Bangladesh (2022), similar focus on healthcare food handlers was observed, though specific distributions differ by region and setting [20, 21]. While the sample size was smaller than that in Al-Akash et al. [22] (*n* = 412), it was adequate for a quasi-experimental design and provides important local data [22]. Demographic characteristics influence baseline knowledge, training reception, and practice adoption. Future research could explore their potential moderating effects on training effectiveness [20].

### Food handlers’ knowledge about food safety

The implementation of the on-the-job training program resulted in an increase in food handlers’ knowledge, which rose from 25.7% to 60%. This improvement was highly significant (*p* < 0.001), demonstrating the program’s effectiveness in imparting vital theoretical knowledge such as food safety hazards, causative organisms, foodborne disease symptoms, food hygiene, and cross-contamination. These findings confirm that the training successfully addressed identified knowledge gaps, fulfilling a primary requirement for safe food handling.

Our results align with a broad body of recent literature, showing that the total knowledge score tripled following the program. Additionally, the number of staff who achieved a ‘good’ rating doubled compared to baseline values. These improvements are supported by Insfran-Rivarola [23], whose systematic review and meta-analysis [23] highlighted the importance of hospital-directed training and registered a significant pooled effect size of 1.24. Similarly, Sinha and Sandhu (2023) substantiated this effect in an Indian tertiary facility, reporting concurrent increases in knowledge among operational food handlers and concluding that educational modules are vital for raising safety understanding [24].

Furthermore, the necessity for such interventions is substantiated by other regional studies. Al Banna [21], in a study of hospital food service staff in Bangladesh, emphasized that functional and job-specific training is essential for advancing staff knowledge [21]. Additionally, Alkhaldi [25] noted that motivated handlers perform better when presented with new, applicable knowledge [26]. Overall, these findings emphasize the recommendation for consistent and mandatory professional growth to address persistent knowledge gaps among staff, ensuring that food handlers possess the intellectual foundation necessary for safe practice.

### Food handlers’ practices toward food safety

Practical adherence increased from 60% to 84.3% (*p* < 0.001), reflecting improvements in handwashing, uniform compliance, contamination prevention, and proper food handling. The food safety training, supplemented with practical demonstrations and redemonstrations, enabled food handlers to not only learn, but also practice, and improve their performance during actual operations. The improvements in practices coincides with the findings of Alkhaldi S [25], in which positive results from training were reported. It instilled confidence in food handlers to engage in practiced food safety [25].

The improvement in adequate practices from 60% to 84.3% in our study is striking and similar to the improvements observed in a study by Al Akash [22], which noted the effectiveness of food safety training programs on practices in food services in hotels and hospitals. According to intervention studies, the education and ongoing supervision of food service staff in certain areas of food service practice are likely to lower food service-related illness and have been documented to do so [22].

Some Egyptian studies conducted in hospital environments substantiate the capacity of systematic food safety instruction to elevate practice of food handlers. Kataya [27] indicated that within the Port Said health-system hospitals, the fraction of staff classified as possessing food safety practices moved from 16.9% to 67.8% and achieved statistical significance [27]. These measures show a marked improvement over the baseline values established at the start of the study.

Consistent with earlier work, Hassan [28] at Ain Shams University hospitals revealed that a structured health education initiative yielded durable and clinically meaningful advancements in practices. Practice scores improved from 8.35 ± 2.06 to 11.40 ± 0.70. Follow-up evaluations carried out immediately, at three months, and at six months documented statistically significant and progressively ascending gains (*p* < 0.001). The gradual enhancement beyond the initial assessment period demonstrates the sustained adoption of safe food-handling competencies when reinforced through strategic training [28]. Although the present study documented outcome measures at a single time point immediately after intervention, the magnitude and direction of improvement correspond closely to Hassan and recent evidence, This confirms that directed training structures are essential for food safety and constitute a sound educational intervention within healthcare food service settings.

A 2019 study of 17 public hospitals in the Gharbia Governorate documented a trend similar to the current study. Baseline measurements initially showed that 76% of food service personnel exhibited inadequate practices (yielding a score of < 50%), while only 2.5% met the threshold for satisfactory competence (≥ 75% threshold). Precision training sessions led to a significant improvement, with the “inadequate” group dropping to 11.2% and the remainder of the cohort achieving scores within the acceptable range (*p* < 0.001). This shift aligns with the trajectory of the current investigation, where acceptable practices increased from 60% to 84.3%, confirming a consistent constructive drift toward hygienic competence [29].

The variation in these results is largely attributed to the initial compliance levels of the participants. In the Port Said studies, including the 2025 investigation, staff began with higher baseline compliance [27]. In contrast, Wahdan [29] began with very low initial scores, which resulted in a more dramatic relative improvement following training. Despite these differing starting points, both datasets definitively confirm that structured educational exercises are capable of significantly propelling the practical implementation of food safety standards [29].

### Correlation between knowledge and practices before and after an on-job training program

A key outcome of this study is the highly significant, direct association between post-training knowledge and practical conduct. While baseline metrics often show a discrepancy between what staff know and what they do, our post-training assessment confirms that this program successfully bridged that gap. This suggests that the combination of on-the-job training and practical application furnished food handlers with the intellectual means to navigate safety issues in their daily responsibilities. These findings achieve the study’s primary aim, confirming that targeted training successfully bridges the gap between theoretical knowledge and daily practice.

This relationship aligns with core principles in public health and behavioral science: effective training is the necessary first step to drive safe and consistent practice. As Alkhaldi [25] noted, motivated food handlers perform better when presented with applicable new knowledge [25]. Furthermore, a 2020 systematic review and meta-analysis confirms that training interventions provide a reliable, positive return on investment [23]. Our results—showing knowledge improvement from 25.7% to 60% and practice enhancement from 60% to 84.3%—reinforce the global consensus that specialized training is a primary driver of hygienic competence.

The trend of our findings is consistent with research by Al Banna [21] and Al-Akash [22], both of whom reported significant improvements in knowledge and practice scores within hospital and hotel food services. Similarly, a 2019 study by Wahdan in Gharbia Governorate [29] and a 2025 study in Port Said by Kataya [27] documented parallel pathways. While the degree of relative improvements varied based on the participants’ initial compliance levels—with Wahdan’s cohort starting at a lower baseline and consequently achieving a more remarkable improvement after the training program—all these studies collectively prove that structured training can lead to an exponential increase in both the knowledge and the practical proficiency of food handlers.

### Strengths of the study

The main strengths of this study are its considerable real-world impact and its thorough methodological approach. Conducting this study in hospital kitchen units made the results applicable to the healthcare sector, as food safety is always critical in this area. Assessments of knowledge and practice were thorough, as validated and reliable questionnaires and observational checklists were used. Training was standardized and carried out in active and participatory formats to ensure learner engagement and accurate delivery of the training content. In addition, the study was framed within ethical parameters, and the results were further corroborated by in-depth quantitative analyses. This further strengthen the results of the study.

### Limitations and future directions

Every study comes with limitations, and this one is no different. There is no control group, and this is a one group pre and post test study. Generalization is difficult for this specific population, and only a convenience sample of 70 food handlers from a single governorate was studied; this population is rapidly losing kitchen food handlers, creating difficulty with follow up. Future studies should incorporate the retention and practice sustained and safe follow up long after training, perhaps 3 months, 6 months, or 12 months down the line.

## Conclusion

This study demonstrates that on-the-job food safety training programs conducted within hospital kitchens positively impact food handlers’ knowledge and practices. This demonstrates the impact of on-the-job training and the importance of having food safety training integrated at food service operations within hospitals as a food safety practice. There is a need for food safety training and assessment activities integrated within food service systems to provide safety for patients, especially vulnerable ones, as they may be at risk of foodborne diseases. Future studies should look at the impact of food safety training integrated at food service systems and how the obtained knowledge and practices may be sustained, while using a control system to facilitate the study.

## Supplementary Information

Supplementary Material 1.

Supplementary Material 2.

Supplementary Material 3.

Supplementary Material 4.

Supplementary Material 5.

Supplementary Material 6.

Supplementary Material 7.

## Data Availability

The datasets generated and analyzed during the current study are not publicly available due to institutional confidentiality and participant privacy requirements. However, anonymized data may be made available from the corresponding author upon reasonable request and subject to approval by the relevant ethics committee.

## Abbreviations

AIDS: Acquired Immunodeficiency Syndrome
CDC: Disease Control and Prevention
CRF: Case fatality rate
HIV: Human immunodeficiency virus
REC: Research Ethics Committee
NCES: National Center for Education Statistics
SPSS: Statistical Package for Social Sciences version
WHO: World Health Organization

## References

[1] Abbas DM, Abed SN, Kadhim RA. The food hygiene practices among workers in restaurants of Wasit Governorate, Iraq. J Pak Med Assoc. 2023;73(9):S88–92.

[2] Lokman U, Akoğlu A. Food allergy knowledge, attitudes, and practices of food handlers working in the five-star hotel kitchens in Turkey. Food Health. 2022;8(1):23–34. 10.3153/FH22003.

[3] Fekadu Y, Kinde MZ, Dagnaw GG, Dessalegn B, Dejene H, Gessese AT, Knowledge. Attitude, and practices on food safety among food handlers working in public food service establishments in Lemi Kura Subcity, addis Ababa, Ethiopia. Biomed Res Int. 2024. 10.1155/2024/2675894. PMID: 38292064; PMCID: PMC10827374. 38292064 10.1155/2024/2675894 PMC10827374

[4] Taha S, Zanin LM, Osaili TM. Studying the perception of leadership styles and food handlers’ hygienic practices in food businesses: the role of commitment and job satisfaction as mediators. Food Control. 2024. 10.1016/j.foodcont.2023.110148. 157,110148.

[5] Salam HH, Eldoom EA, Ali FF. The effect of training of food handlers in hospitals’ kitchens in Khartoum State, Sudan. Magna Scientia Adv Biology Pharm. 2021;03(01):001–12. 10.30574/msabp.2021.3.1.0028.

[6] Guennouni M, Admou B, Bourrhouat A, El Khoudri N, Zkhiri W, Talha I, Hazime R, Hilali A. Knowledge and practices of food safety among health care professionals and handlers working in the kitchen of a Moroccan university hospital. J Food Prot. 2022;85(4):676–85. 10.4315/JFP-21-305. 10.4315/JFP-21-305 35051278

[7] Malavi DN, Abong GO, Muzhingi T. Effect of food safety training on behavior change of food handlers: A case of orange-fleshed sweet potato purée processing in Kenya. Food Control. 2021;119:107500. 33390669 10.1016/j.foodcont.2020.107500 PMC7607239

[8] Alsultan SB, Allowaymi SS, Alshammari GM, Alrasheed A. Cross-Sectional investigation of the awareness and practices of food safety among food service catering staff in Riyadh City hospitals during the coronavirus pandemic. Healthcare. 2023;11(8):1134. 10.3390/healthcare11081134. 37107968 10.3390/healthcare11081134 PMC10137720

[9] Hafez FE, Sobeh DE, Ibrahim AM. Food handlers' knowledge, attitude, and practices about safe and hygienic food in Egyptian government hospitals. Egyptian J Health Care. 2022;13(2):1546–59.

[10] Abdellhakeem AA, Feyza B, Hekmat A. Food safety knowledge among food handlers in hospitals of Jordan. Food Sci Technol. 2021;9(2):17–30. 10.13189/fst.2021.090201.

[11] Boakye NA, Boateng NA, Hagan JA. Assessing the hygienic state of hospital kitchens in the Sekondi – Takoradi metropolis of Ghana. Eur J Hospitality Tourism Res. 2016;3(3):1–9.

[12] Ali S, Aslam R, Arshad MI. Meat-borne bacterial pathogens. Veterinary Pathobiology Public Health. 2021;217. 10.47278/book.vpph/2021.018.

[13] Faour-Klingbeil D, Todd CD. Prevention and control of foodborne diseases in Middle-East North African countries: review of National control systems. Int J Environ Res Public Health. 2019;17(1):70. 10.3390/ijerph17010070. PMID: 31861843; PMCID: PMC6982137. 31861843 10.3390/ijerph17010070 PMC6982137

[14] Abdalfatah WH, Osman SR. Evaluation of an educational program concerning food safety for food services employees in Assiut university Restaurants, Assiut Governorate. Assiut Sci Nurs J. 2020;8(21):167–75.

[15] Mohd-Yusof AM, Rahman NA, Haque M. Knowledge, attitude, and practice toward food poisoning among food handlers and dietetic students in a public university in Malaysia. J Pharm Bioallied Sci. 2018;10(4):232–9. 10.4103/JPBS.JPBS_141_18. 30568381 10.4103/JPBS.JPBS_141_18 PMC6266644

[16] York WT, MacAlister D. Training and Development, Hospital and Healthcare Security (Sixth Edition), (2015); Chap. 10 -2015, Pages 225–259, ISBN 9780124200487. 10.1016/B978-0-12420048-7.00010-6

[17] Bilge N, Demir P. Evaluation of food safety knowledge among food handlers. Van Vet J. 2019;30(1):7–12.

[18] Egypt National Food Safety Authority. Law No. 1 of 2017: Establishing the National Food Safety Authority. The Official Gazette, Issue No. 1 bis (C). 2017.

[19] The Food and Agriculture Organization of the United Nations (FAO)/World Health Organization WHO Assuring Food Safety Quality. Guidelines for strengthening national food systems. 2016; Available at https://www.fao.Org/docrep/006/y8705e/y8705e00.HTM.

[20] Bou-Mitri C, Mahmoud D, El Gerges N, Jaoude MA. Food safety knowledge, attitudes, and practices of food handlers in Lebanese hospitals: A cross-sectional study. Food Control. 2018;94:78–84. 10.1016/j.foodcont.2018.06.032.

[21] Al Banna MH, Khan MSI, Rezyona H, Seidu A-A, Abid MT, Ara T, Kundu S, Ahinkorah BO, Hagan JE Jr., Tareq MA, Begum MR, Chowdhury MFT, Schack T. Assessment of food safety Knowledge, attitudes and practices of food service staff in Bangladeshi hospitals: A Cross-Sectional study. Nutrients. 2022;14(12). 10.3390/nu14122540. 10.3390/nu14122540 PMC9227153 35745271

[22] Al-Akash H, Abu Arrah A, Bhatti F, Maabreh R, Abu Arrah R. The effect of food safety training program on food safety knowledge and practices in hotels’ and hospitals’ food services. Ital J Food Safety. 2022 Feb. 22 2025 Aug. 19;11(1). Available from: https://www.pagepressjournals.org/ijfs/article/view/9914. 10.4081/ijfs.2022.9914 PMC8883829 35284344

[23] Insfran-Rivarola A, Tlapa D, Limon-Romero J, Baez-Lopez Y, Miranda-Ackerman M, Arredondo-Soto K, Ontiveros S. A systematic review and Meta-Analysis of the effects of food safety and hygiene training on food handlers. Foods. 2020;9(9):1169. 10.3390/foods9091169. PMID: 32854221; PMCID: PMC7555000. 32854221 10.3390/foods9091169 PMC7555000

[24] Sinha S, Sandhu NK. Effectiveness of educational intervention on KAP of food handlers and barriers to implementation of food safety: a cross-sectional comparative study. Int J Community Med Public Health. 2023;10(6):2192–6. 10.18203/2394-6040.ijcmph20231702.

[25] Alkhaldi S, Hod Z, Isa ZM, Idris IB, Karim F. The Impact of Food Safety Training Programs on Knowledge, Attitude, and Practice on Food Safety Among Migrant Workers – A Review. Nutr Food Sci. 2025; 13(2). Available from: https://bit.ly/4kGOq9G.

[26] Teffo LA, Tabit FT. An assessment of the food safety knowledge and attitudes of food handlers in hospitals. BMC Public Health. 2020;20:311. 10.1186/s12889-020-8430-5. 32164674 10.1186/s12889-020-8430-5 PMC7069208

[27] kataya lf. Health educational program for food handlers about food safety in Port said hospitals. Port Said Sci J Nurs. 2025;12(1):305–35. 10.21608/pssjn.2025.367951.1351.

[28] Hassan SF, ElBagoury LS, Amin GEA, Meky FA, Galmal DA. Development and assessment of health education program regarding food safety among food handlers at Ain Shams university hospitals, Cairo, Egypt. Int J Health Sci. 2022;6(S3):511–22. 10.53730/ijhs.v6nS3.5359.

[29] Wahdan IH, Gad ZM, Ihab M, Habib IM, Elshabasy OA. Effect of an educational program on food safety practices in food Preparation and handling procedures in governmental hospitals of an Egyptian Governorate. J High Inst Public Health. 2019;49(2):90–6.

