# Supplementary material for: On-job training program for food handlers about food safety standards
Source: BMC Public Health. 2026 Mar 11;26:1241. doi: 10.1186/s12889-026-26228-4 (PMC13085662; doi:10.1186/s12889-026-26228-4)

Supp. Figure (1): Percentage distribution of the studied food handlers according to their sociodemographic characteristics (N=70).

Supp. Figure (1.A): Percentage distribution of the studied food handlers according to their gender (N=70).

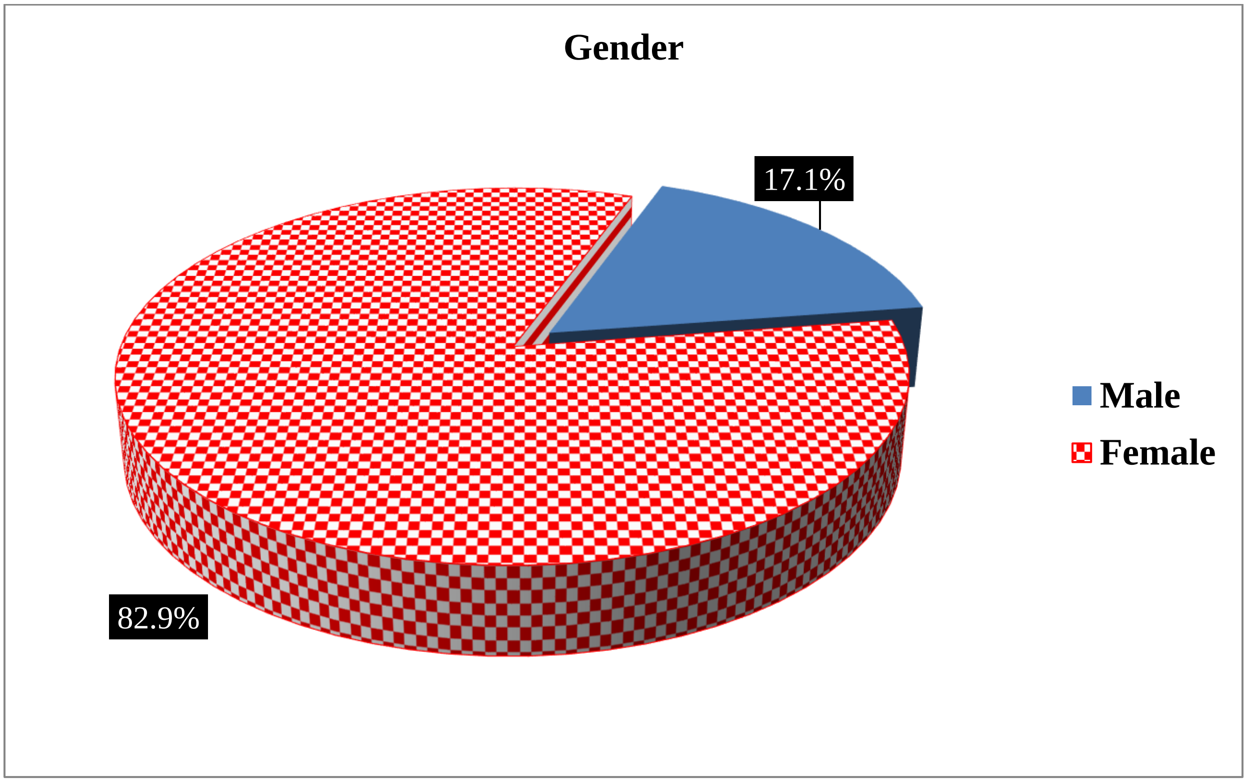

Supp. Figure (1.B): Percentage distribution of the studied food handlers according to their years of experience (N=70).

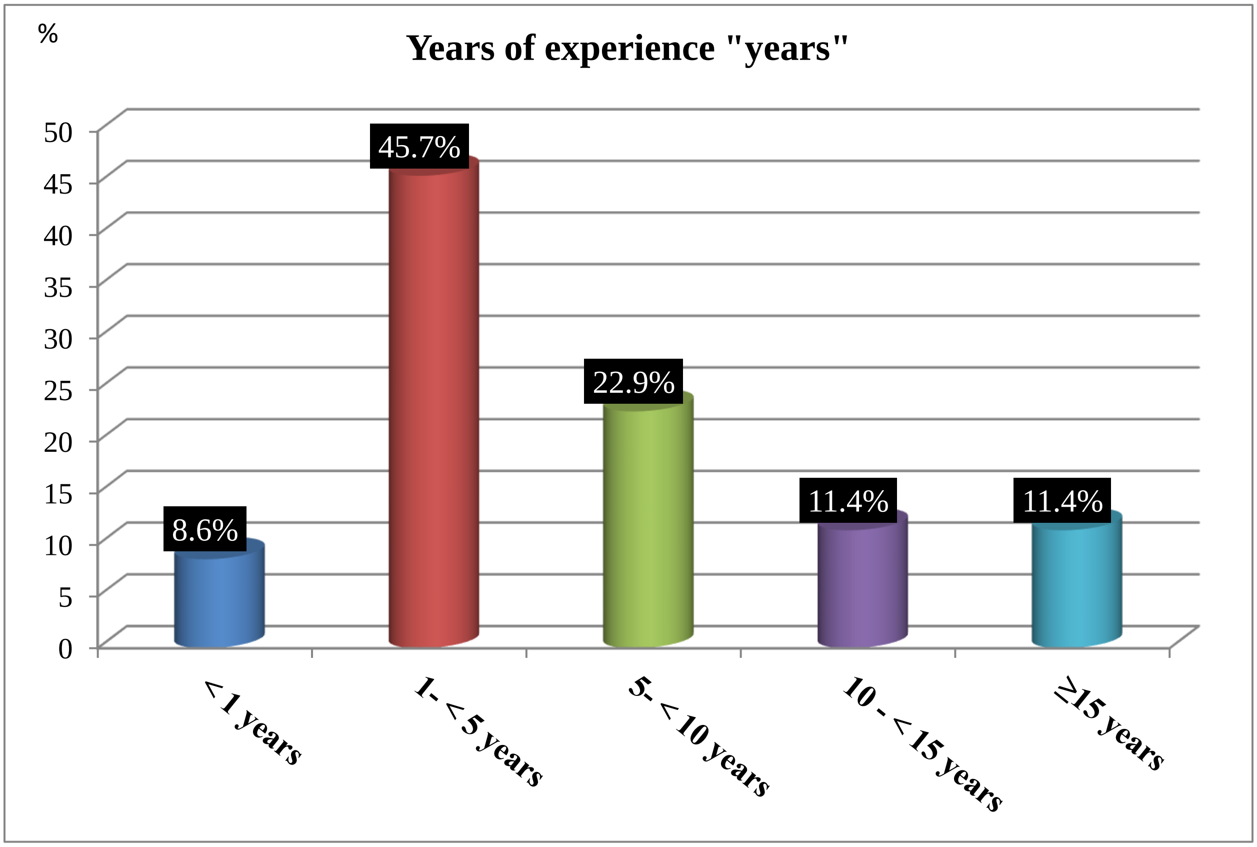

Supp. Figure (1.C): Percentage distribution of the studied food handlers according to their educational qualifications (N=70).

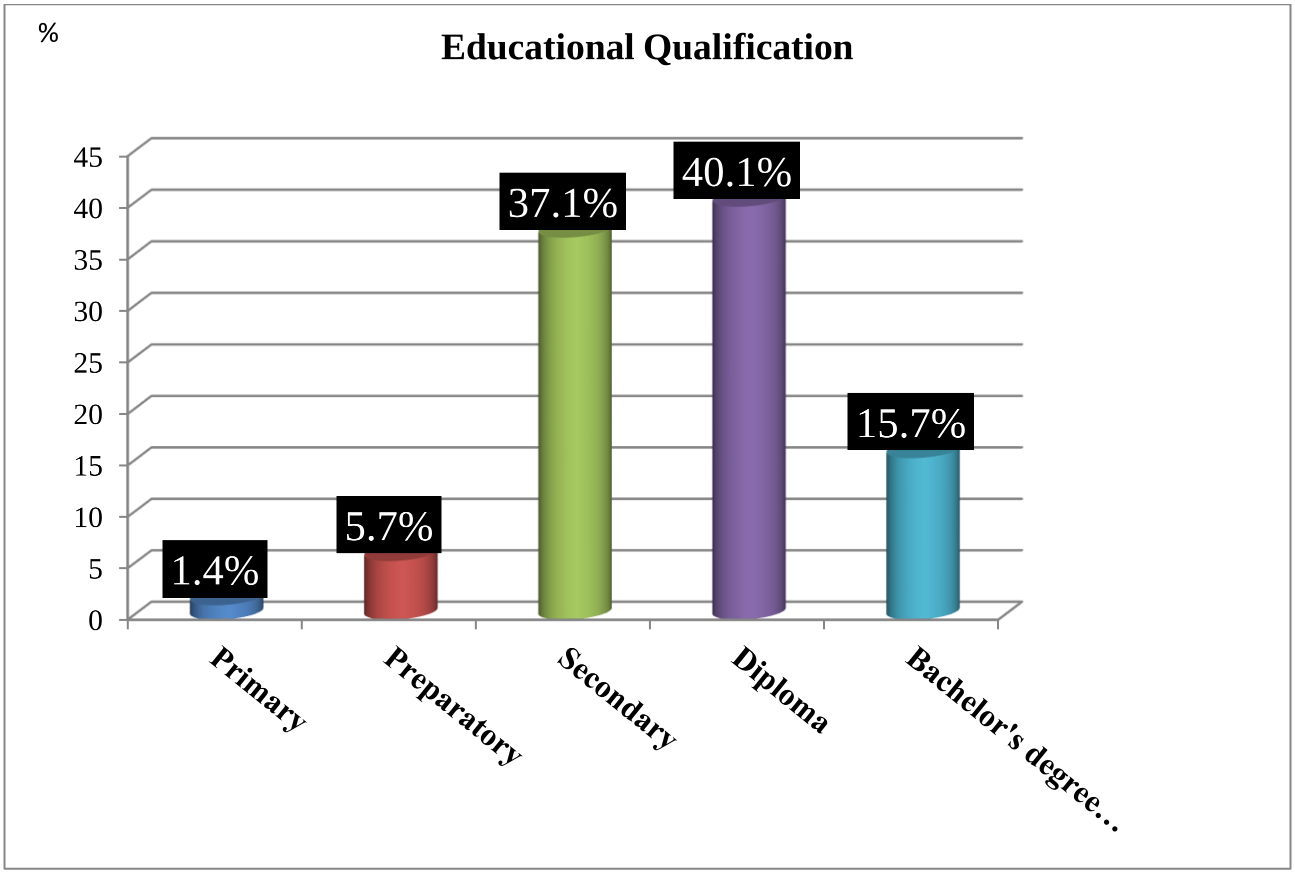

Supp. Figure (1.D): Percentage distribution of the studied food handlers according to their working activities (jobs) (N=70).

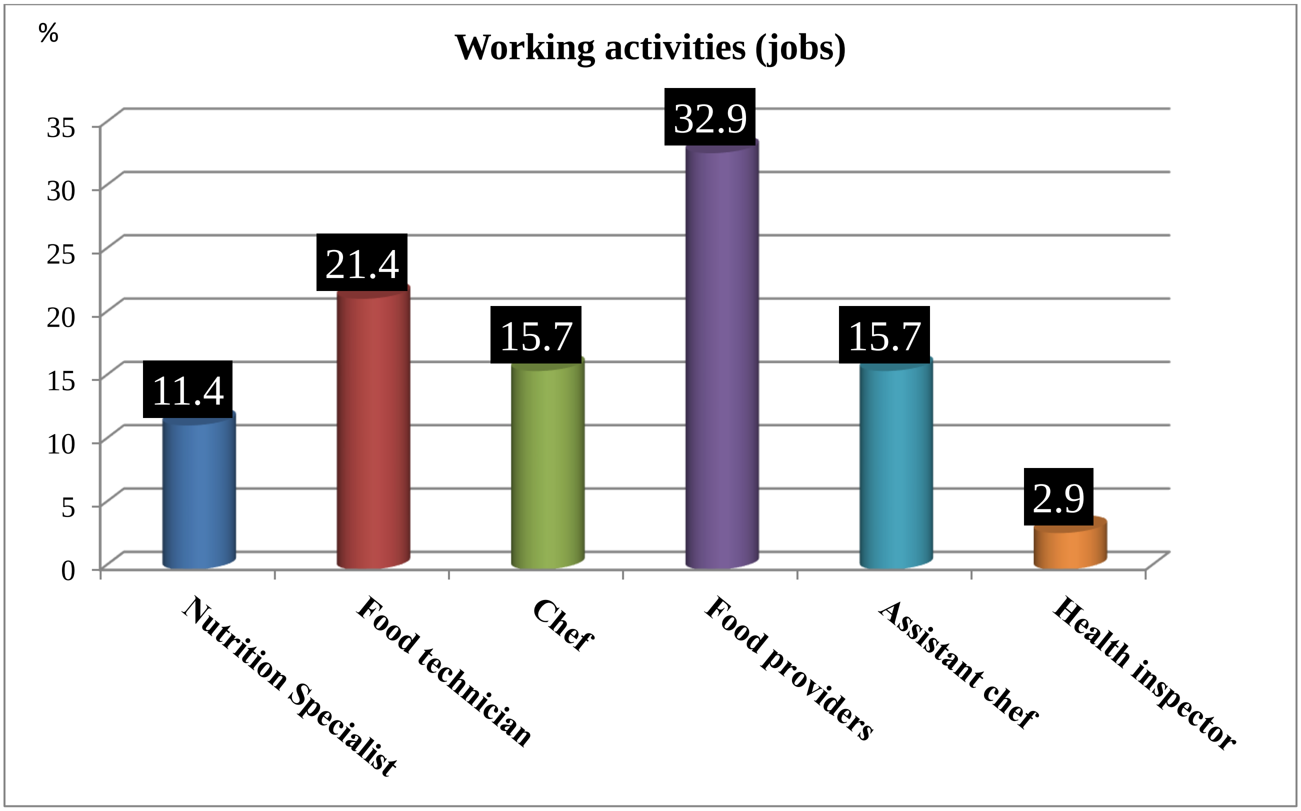

Supplement: Supplementary file 1 — Supplementary Material 1. [file 12889_2026_26228_MOESM1_ESM.pdf]
