# Supplementary figures and images for: On-job training program for food handlers about food safety standards
Source: BMC Public Health. 2026 Mar 11;26:1241. doi: 10.1186/s12889-026-26228-4 (PMC13085662; doi:10.1186/s12889-026-26228-4)

**Supp. Figure (2): Distribution of food handlers according to their health certificates: N=70**

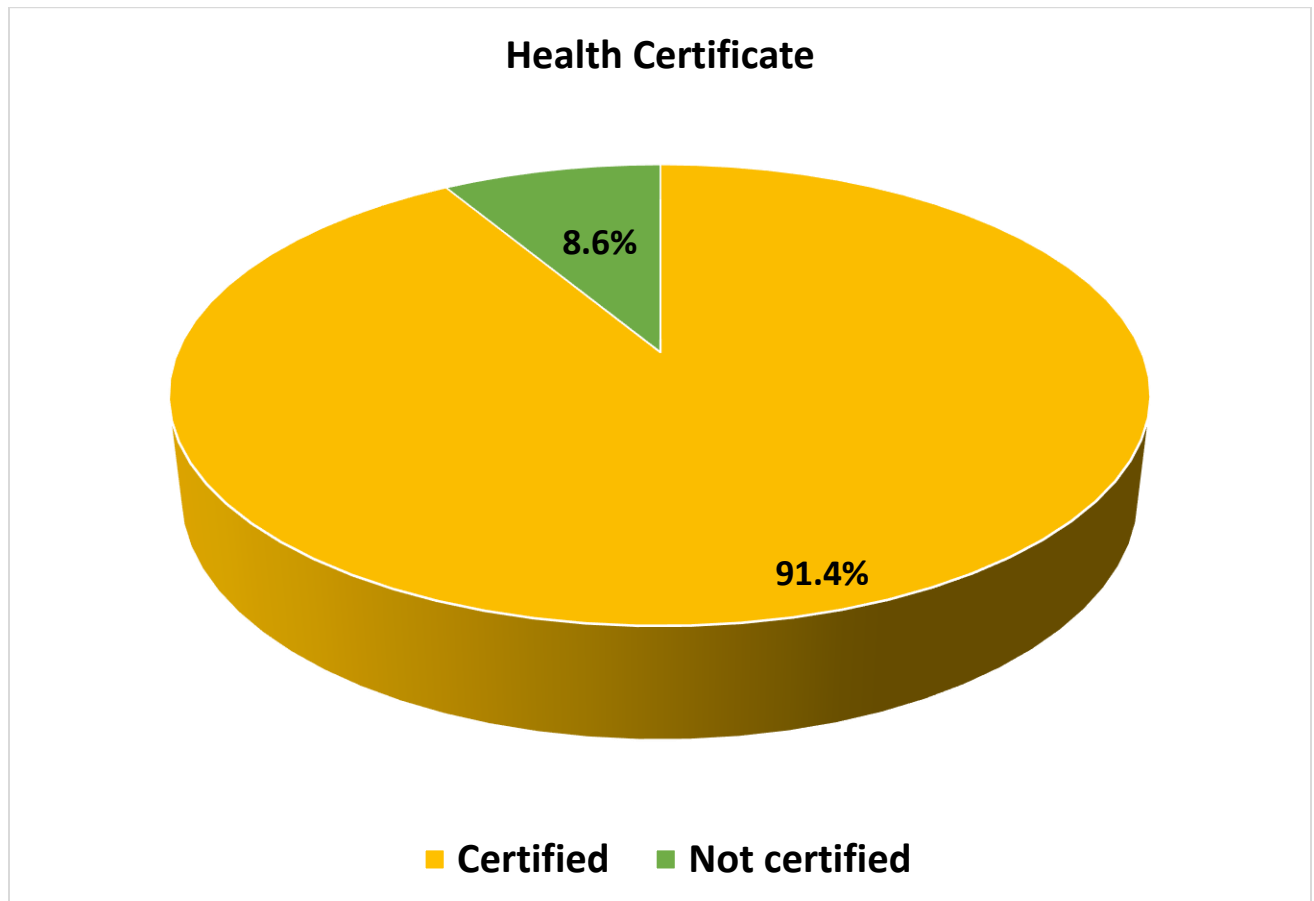

Supplement: Supplementary file 2 — Supplementary Material 2. [file 12889_2026_26228_MOESM2_ESM.pdf]

**Supp. Figure (3): Distribution of food handlers according to their vaccination's status: N=70**

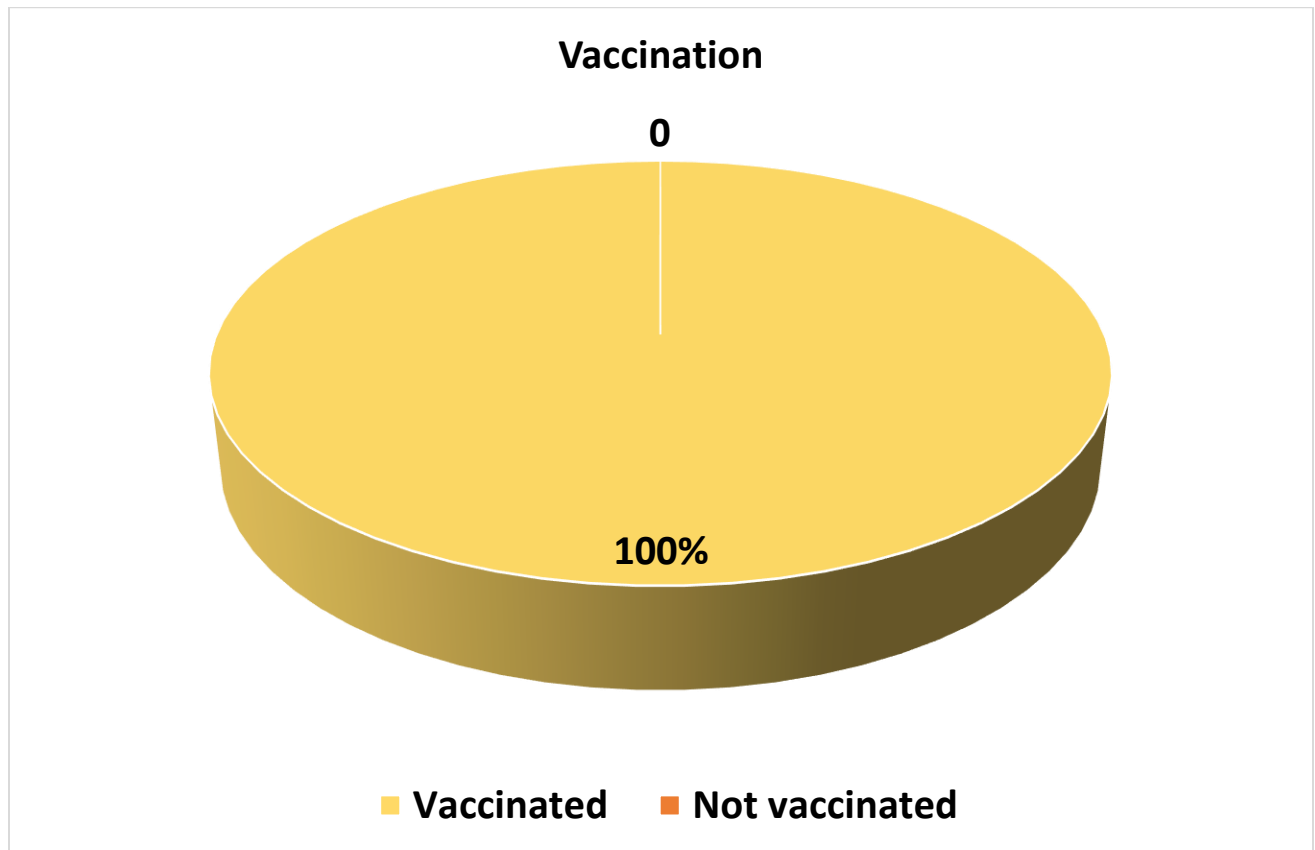

Supplement: Supplementary file 3 — Supplementary Material 3. [file 12889_2026_26228_MOESM3_ESM.pdf]
