# Supplementary material for: On-job training program for food handlers about food safety standards
Source: BMC Public Health. 2026 Mar 11;26:1241. doi: 10.1186/s12889-026-26228-4 (PMC13085662; doi:10.1186/s12889-026-26228-4)

Supp. Figure (4): Frequency distribution of the studied food handlers according to their total level of knowledge about food safety standards "Pre and Post program implementation" (N=70).

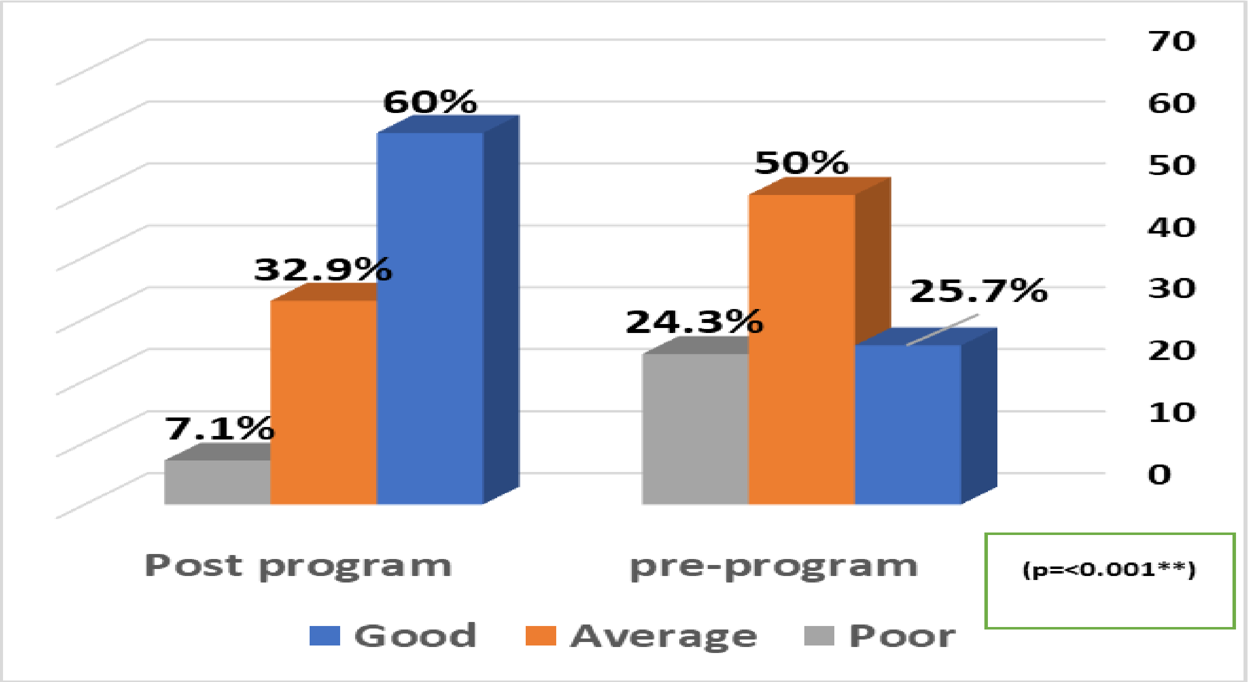

Supplement: Supplementary file 4 — Supplementary Material 4. [file 12889_2026_26228_MOESM4_ESM.pdf]
