# Supplementary material for: On-job training program for food handlers about food safety standards
Source: BMC Public Health. 2026 Mar 11;26:1241. doi: 10.1186/s12889-026-26228-4 (PMC13085662; doi:10.1186/s12889-026-26228-4)
Supplement: Supplementary file 6 — Supplementary Material 6. [file 12889_2026_26228_MOESM6_ESM.pdf]

Questionnaire for food Handlers

Dear Food handlers

This study aims to In-service Training Program for Food Handlers about Food Safety Standards at General Authority of Health Care Agency

Please mark (☐) in front of each of the following items in the column that expresses your opinion.

We thank you for your kind cooperation.

Serial number: ....

<u>First: Personal data:</u>

Name: ……………………………… (optional)

Gender: ☐ Male ☐ Female

Age: …………

Years of experience: ....

Educational Qualification:

☐ Primary ☐ Preparatory ☐ Secondary
☐ Diploma ☐ Bachelor's degree or higher

Working activity (job):

☐ Nutrition Specialist
☐ Food technician
☐ Chef
☐ Food providers
☐ Assistant chef ☐ Health inspector

Place of work: …………

<u>Second: Other data:</u>

Food safety training: Have you received food safety training courses?

☐ Yes ☐ No

Health Certificate:

Do you have a valid and updated health certificate to deal in the field of food?

☐ Yes ☐ No

Vaccinations:

- Have you had any vaccinations?

   Yes   No

If yes, please specify mention the name of the vaccination……….

Assessment of Food Handlers' Knowledge about Safe Food Handling

Items	Correct	Incorrect
**1. Meaning of Food safety is:**		
A- The food should taste and smell good		
b- The food must be free from chemical, biological and physical hazards that may lead to disease or even death for the consumer		
C- The necessary conditions and measures during food production, processing, storage, distribution and preparation to ensure its safety and suitability for human consumption.		
d- The right definition only (b, c)		
e- All of the above All of the above		
**2. The importance of food safety is:**		
a- Protect customers from food poisoning and foodborne diseases that may affect human life		
b- Eat a healthy food		
c- Eat food that tastes and smells good		
e- All of the above		
**3. The types of hazards that make food unsafe are:**		
a- Biological, physical and chemical hazards and allergens		
b- Bacterial, food and global risks		
c- Fungi, viruses and parasites		
d- Allergens, disinfectants and pollutants		
e- All of the above		
**4. Chemical pollutants can include the following:**		
a- Pesticides		
b- The dirt		
c- Toothpicks		
d- Broken glass		
e- All of the above		
**5. The basic requirements for food safety are:**		
a- Hygiene: Personal hygiene and surface cleanliness		
b- Separation: Preventing cross contamination		
c- Good cooking: Cooking to the right temperature		

Items	Correct	Incorrect
d-Cooling: Instant cooling		
e- All of the above		
**6. The instructions that must be followed to ensure food safety are:**		
a - You should not rely entirely on the smell, taste, or shape of the food. Sometimes these foods seem edible, but in reality, they are not.		
b- The presence of bacteria or their toxins does not change the taste, taste, smell or shape in many cases.		
C- There are many cases of food poisoning that occur daily, and some of them may be due to tasting food that may not be safe.		
d- All of the above		
**7. The best way to destroy any harmful germs that may be present in food is to:**		
a. Adding salt to the food		
b. Keep foodstuffs at room temperature		
c. Cook food to the right temperature		
d. Keep food below 5°C at all times		
e. All of the above		
**8. Which temperature do you maintained for potentially hazardous food that has been cooked and needs to be reheated:**		
a. Re-heat to 54°C in a steam table or other hot holding equipment		
b. Re-heat at 63°C or above		
c. Re-heat slowly in the oven to 68°C, stirring at least twice.		
d. Re-heat quickly to 75°C or above		
**9. The best way you use to control pests and animal is to:**		
a. Apply pesticide everyday		
b. Just sweep the floor		
c. Pour chlorine in the sink drains		
d. Keep the establishment and garbage area clean, and eliminate hiding places and routes of entry		
e. Control cockroaches, mice, flies and other pests periodically		

Items	Correct	Incorrect
**10. Where do you store cleaning items and sanitizers:**		
a. Away from food items or clean equipment and utensils		
b. With equipment's and cleaning utensils		
c. On the shelf above food and utensils		
d- All of the above		
**11. How many times can you reheat leftovers:**		
a. As many times as you like		
b. Twice		
c. Four times		
d. You should only reheat leftovers once		
**12. The safest way you use to thaw (defrost) food is to:**		
a. Keep container at room temperature		
b. Sink with hot running water		
c. Sink at room temperature over night		
d. Keep in the refrigerator		
**13. Frozen food storage is generally operated at temperature of:**		
a. 0 °C		
b. -15 °C		
c- -18 °C		
c. -50 °C		
d. -60 °C		
e. All of the above		
**14. You should Keep the refrigerator temperature at or below:**		
a. 6° C		
b. 4° C		
c. 8° C		
d. 5° C		
e. All of the above		
**15. What is the meaning of food allergies**		
a. An immunologic reaction resulting from the ingestion, contact or inhalation of a food or food additive		
b. An adverse reaction to a food in which the body's immune system is not involved		

Items	Correct	Incorrect
c. A metabolic food disorder where food borne substances interfere with normal metabolic processes		
d. All of the above		
**16. Which of the following not considered as a major food allergen**		
a. Milk		
b. Egg		
c. Poultry		
d. Peanuts		

Assessment of Food Handlers' Knowledge about Food Hygiene

Items	Correct	Incorrect
**1. Food hygiene include the following procedure:**		
a. Hand washing		
b. Cook food in a suitable temperature		
c. Wear personal protection equipment while distributing food		
**d- All of the above**		
**2. Food hygiene is necessary for:**		
a. Avoiding food poisoning		
b. Preventing foodborne diseases		
c. All of the above		
**3. Hand washing should be done in the following occasions:**		
a. Before handling food		
b. After handling food		
c. Before wearing gloves		
d. In between preparations		
e. All of the above		
**4. How frequently do you wash your hands**		
a. Every 30 minutes		
b. When your supervisor tells you		

Items	Correct	Incorrect
c. Each time your hands or gloves become contaminated		
d. Before and after any procedure		
d. All of the above		
**5. What is time duration for soap water hand washing**		
a. 20-30 sec		
B. 2 minutes		
c. 40-60 sec		
**6. Hands must be dried after washing in order to:**		
a. Prevent dripping of water		
b. Prevent germs and bacteria which get spread with wet hand		
c. Hold the utensils properly		
d. only (a and b)		
**7. The correct way to dry hands after washing them is:**		
a. Use a cotton wool		
b. Just shake excess water away		
c. Use an air dryer		
d. Use a paper towel		
e. All of the above		
8. Precautions to prevent foodborne diseases are:		
a- Serving food without washing hands		
b- Washing hands, wearing gloves, and keeping foodstuffs at the correct temperature		
C- Washing hands once a day		
D - Spray pesticides on the kitchen floor once a month		
**9. The correct place to wash your hands is:**		
a. Any sink that is free and accessible		
b. Specific sink for washing hands		
c. Hand-washing basin or a dish-washing basin		
d-All of the above		
**10.Which of the following you ever substitute for proper hand washing:**		
a. Hand sanitizers		
b. Tissue paper		

Items	Correct	Incorrect
c. Wearing loves		
d. You never substituted proper hand washing		
**11. The difference between washing and sterilizing is:**		
a .Washing removes contamination and sanitizing destroys microorganisms.		
b .Washing makes things look clean and sanitizing makes them smell good.		
d. There is no difference		

Assessment of Food Handlers' Knowledge about Food Borne Diseases

Items	Correct	Incorrect
**1. What is the meaning of Food borne diseases:**		
a. Any disease caused by consuming foods or beverages contaminated with harmful pathogens - such as bacteria, viruses, and fungi — or their toxins		
b. Any disease caused by contact with contaminated surface		
c. Any disease caused by contact with sick person		
**2. What are the causes of food poisoning:**		
a. Not washing hands properly		
b. Serving contaminated food		
c. Touching ready to eat food with bare hands		
d. All of the above		
**3. What are the signs and symptoms of food poisoning:**		
a. Vomiting, Nausea		
b. Stomach ache		
c. Diarrhea		
d. All of the above		
**4. What are the Factors affecting the growth and reproduction of bacteria on foods**		
a. Heat		
b. Time		
c. Humidity		
d. Acidity		
e. Oxygen		
f. Food		
f. All of the above		
**5. Food should not be left at room temperature more than:**		
a - Two hours		
b- 10 hours		
c- 5 hours		
d. 6 hours		
A - None of the above.		

Items	Correct	Incorrect
**6. Food handlers can spread food borne diseases by:**		
a- Not washing hands		
b- Presence of a skin injury		
C- Nasal discharge or respiratory illness (sneezing/coughing)		
d- Handling food or utensils during diarrhea, fever, vomiting or stomach cramps		
e- Poor personal hygiene		
f- All of the above		
**7. People most susceptible to food poisoning**		
a- Children		
b- The elderly		
c- Pregnant women		
d- People with weak immunity		
e- All of the above		
**8. The infection is transmitted from the food handler to other people in the following cases:**		
a-When one of the food handlers is infected or carrier of a foodborne disease		
b-When one of the food handlers has a wound or a cut on his hands that contains microbes and handles the food with exposed hands.		
c- When one of the food handlers has dealt with/been in contact with a person infected with a foodborne disease		
D- When one of the food handlers has touched anything that might contaminate his hand and not wash it before handling the food		
e- When one of the food handlers has symptoms of diarrhea / vomiting / fever / yellowing of the skin and eyes.		
f- All of the above		
**9. The main sources of food contamination are:**		
a- The environment		
b- Pests.		
c- Poor hygiene of utensils and buildings		
d- Rubbish.		

Items	Correct	Incorrect
e- Dealing with the food itself		
f- All of the above		
**10. The main causes of foodborne illnesses are:**		
a- Poor personal hygiene		
b- Unsuitable temperatures for preserving food		
c- Unsuitable temperatures for cooking		
d- Contaminated equipment		
e- Food from unsafe sources		
f- All of the above		

Assessment of Food Handlers' Knowledge about Practices that Prevent Contamination

Items	Correct	Incorrect
**1. In order to prevent food, contaminate through your own clothes, you need to:**		
a .Store your personal belongings and clothing in the food storage area in the corner.		
b. Change in the bathroom.		
c. Store personal belongings and clothing in a locker in changing room		
d. All of the above		
**2. Which of the following is important to prevent food poisoning:**		
a. Thoroughly wash and dry hands properly		
b. Never cough or sneeze over food or where food is prepared or stored		
c. Covering your hair.		
d. All of the above		
**3. What do you do before handling food item:**		
a. I wear gloves		
b. I wash my hands thoroughly with warm water and soap for at least 40 seconds		

Items	Correct	Incorrect
c. Take the clean dishes out of the dishwasher		
d. Rinse my hands quickly		
**4. If you have a bad cold, fever, and diarrhea you need to:**		
a. Wash your hands before work		
b. Take medicine before going to work.		
c. Take adequate rest at home		
d. Not handle food until he receives medical clearance		
e. All of the above		
**5. Which of the following symptoms make you away from work in the kitchen:**		
a. Vomiting		
b. Fever		
c. Diarrhea		
d. All of the above		
**6. If you have a cut on your hand, you must:**		
a. Stay at home		
b. Continue working until bleeding stop.		
c. Stop the bleeding, cover it with bandage and wear gloves		
d. Keep your hand elevated to stop bleeding		
**7. The important measure to keep harmful germ away from food is to:**		
a. Wash your hands thoroughly and frequently and use gloves when necessary		
b. Do not touch anything other than food		
c. Remove all jewelers prior to working with food		
d. Use of hair net/caps before handling food		
e. All of the above		
**8. The best way you use to control contamination is to:**		
a. Keep the floor and walls clean		
b. Cool down hot food quickly		
c. Wash hands as often as necessary and do not touch ready-to-eat food with bare hands		
d. Keep hot food hot and cold food cold		

Items	Correct	Incorrect
e. All of the above		
**9. Are you allowed to work in the kitchen, if you have a contagious illness:**		
a. Yes		
b. I am allowed to work in the kitchen according to the type of infectious disease.		
c. Never allowed		
d. Allowed only if no one tell me that I am sick		
**10. You cannot touch the following foodstuffs with your hands without wearing gloves:**		
a. Ready-to-eat food		
b. Spoiled food		
c. All of the above		
**11. Which of the following jewelry is not acceptable for you to wear in the kitchen:**		
a. Necklace		
b. Arm ring		
c. Bracelet		
d. None of the above		
**12. When you prepare cooked and raw foods you need to take the following precautions:**		
a. I can use the same kitchen utensils to prepare cooked and raw food		
b. I can use separate kitchen utensils to prepare cooked and raw food.		
c. There is no need to take precautions when preparing cooked and raw foods.		
d. I only take precautions when asked by the supervisor.		
