# Supplementary material for: On-job training program for food handlers about food safety standards
Source: BMC Public Health. 2026 Mar 11;26:1241. doi: 10.1186/s12889-026-26228-4 (PMC13085662; doi:10.1186/s12889-026-26228-4)
Supplement: Supplementary file 7 — Supplementary Material 7. [file 12889_2026_26228_MOESM7_ESM.pdf]

An observational checklist

A. Observational checklist to of food handlers' practice about food hygiene.

Items	Met	Not met
**Wear work clothes:**		
1. All staff wearing a head covering.		
2. All staff wear slip-resistant shoes.		
3. All staff wear their uniform.		
**Hand wash**		
1. All staff wash their hands before starting work.		
2. All staff wash their hands after using bathroom.		
3. All staff wash their hands before and after dealing with ready-to- eat food.		
4. All staff wash their hands before and after handling raw.		
5. All staff wash their hands after sneezing and coughing.		
6. All staff wash their hands after touching the hair, nose and ear.		
7. All staff wash their hands before wearing gloves.		
8. All staff wash their hands properly.		
**Prevention of food contamination**		
1. All staff maintain a smoke-free environment in the kitchen		
2. All staff consistently refrain from wiping their hands-on personal clothes.		
3. All staff's clothes are clean		
4. All staff take proper precautions when coughing or sneezing		
5. All staff dry their hands with paper towels after washing.		
6. All staff consistently refrain from chewing gum in the kitchen.		
7. All staff wear gloves during serving food		
8. All staff consistently refrain from touching their hair during work tasks.		
9. All staff refrain from touching their hair during work to maintain hygiene standards.		
10. All staff consistently refrain from eating in the kitchen.		

Items	Met	Not met
11. All staff exhibit no signs of foodborne illnesses in the workplace		
12. All staff remove jewelry and watches before beginning work.		
13.All staff use separate tools for food preparation, such as knives and cutting boards		
14.All staff use a single-use spoon to taste food.		
15.All staff maintain short and clean fingernails.		
16. All staff with abrasion or cut on their hands wear appropriate protective coverage		

B. Observational checklist to assess food handler's practice regarding the course of work in the kitchen.

Items	Met	Not met
**Receiving:**		
1. Food transport vehicles comply with all transportation requirements.		
2. All types of food received, along with their condition, are properly recorded.		
3. No food items are placed on the floor upon receipt.		
4. Received foods are immediately transported to designated storage areas.		
5. Meat is received by a committee that includes a veterinarian.		
**Preparation:**		
1. Food is washed in a designated area.		
2. Vegetables and fruits undergo proper washing and disinfection.		
3. Each type of food is prepared in a separate area to prevent cross-contamination.		
**Thawing:**		
1. Food thawing follows proper methods.		
2. Frozen foods are covered while defrosting.		
3. Defrosted food is used within one day of being removed from the freezer.		
4. Food is not refrozen after defrosting		
**Cooking:**		
1. Proper temperatures are maintained during cooking.		
2. All food is covered after cooking.		
3. Cooked food is not left at dangerous temperatures for more than two hours		

Items	Met	Not met
**Distribution:**		
1. Food distribution units are clean.		
2. Ready-to-eat food is preserved at the appropriate temperature.		
3. Meals are covered during distribution.		
4. Drinks are covered during distribution.		
